# Discovery of Bladder Cancer-related Genes Using Integrative Heterogeneous Network Modeling of Multi-omics Data

**DOI:** 10.1038/s41598-017-15890-9

**Published:** 2017-11-15

**Authors:** Chen Peng, Ao Li, Minghui Wang

**Affiliations:** 1 School of Information Science and Technology, University of Science and Technology of China, Hefei, AH230027 China; 2 Institute of Machine Learning and Systems Biology, College of Electronics and Information Engineering, Tongji University, Shanghai, 201804 P.R. China; 3 Centers for Biomedical Engineering, University of Science and Technology of China, Hefei, AH230037 China

## Abstract

In human health, a fundamental challenge is the identification of disease-related genes. Bladder cancer (BC) is a worldwide malignant tumor, which has resulted in 170,000 deaths in 2010 up from 114,000 in 1990. Moreover, with the emergence of multi-omics data, more comprehensive analysis of human diseases become possible. In this study, we propose a multi-step approach for the identification of BC-related genes by using integrative Heterogeneous Network Modeling of Multi-Omics data (iHNMMO). The heterogeneous network model properly and comprehensively reflects the multiple kinds of relationships between genes in the multi-omics data of BC, including general relationships, unique relationships under BC condition, correlational relationships within each omics and regulatory relationships between different omics. Besides, a network-based propagation algorithm with resistance is utilized to quantize the relationships between genes and BC precisely. The results of comprehensive performance evaluation suggest that iHNMMO significantly outperforms other approaches. Moreover, further analysis suggests that the top ranked genes may be functionally implicated in BC, which also confirms the superiority of iHNMMO. In summary, this study shows that disease-related genes can be better identified through reasonable integration of multi-omics data.

## Introduction

Bladder cancer (BC) is a common malignant tumor, which is characterized by poor clinical outcome and frequent recurrence^1–3^. This malignancy is described as genetic disease, which is caused by multi-step accumulation of both epigenetic and genetic factors^3^. Although the treatment is greatly advanced, the prognosis of BC remains poor^4^. Therefore, there is an urgent need for researchers to identify genes related to BC, which can help uncover the mechanisms underlying this cancer and make an improvement in its diagnosis and therapy.

The identification of BC-related genes is an issue of prioritization of disease-related genes. The most common way to address this issue is to evaluate the similarities between known disease-related genes and given candidate genes. Various information can be used to calculate these similarities such as sequence^5–7^, functional annotation^8^ and protein-protein interactions (PPIs)^9–13^. Two famous methods are proposed by using PPIs: random walk^14^ and PRINCE^15^. In^14^, Kohler *et al*. prioritize genes related to disease by calculating the similarities of genes in PPI networks based on random walk analysis. In this process, the walkers that have the same initial probabilities transit to randomly selected neighbors from known disease-related genes^14^. Later, Vanunu *et al*. introduce prior information into the prioritization function and propose PRINCE^15^. Despite the great success of these two methods in identifying disease-related genes, they only employ the information of PPIs, which cannot reflect the unique relationships between genes under certain disease condition. Especially, rapid development of DNA sequencing technology promotes large projects such as ICGC^16^ and TCGA^17^, which produce enormous experimental data of different cancer in several omics including epigenomics, genomics and transcriptomics^16,17^. For example, more than 200 genomic rearrangements and segmental alterations per sample are detected in BC according to TCGA^17^. Analysis of these molecular aberrations in multiple omics can be very helpful for the improvement in diagnosis, treatment and prevention of cancer.

There are already many researches that use disease data of genes in multiple omics to identify BC-related genes. For example, Reinert *et al*. identify novel genes with tumor-specific differential methylation, which are shown to be promising cancer markers for early detection of BC, through a mapping of methylome^18^. Similarly, Zaravinos *et al*. find 17 differentially expressed genes that may be putative markers of BC by using genome microarrays^19^, *i*.*e*., gene expression data^20–22^. Besides, Zhang *et al*. suggest that susceptibility of BC can be predicted by the copy number variation (CNV) of GSTM1 by using multivariate logistic regression^23^. Although these studies can discover BC-related genes by making use of the disease data of one certain omics, a methodological limitation is the absence of efficient integration of different high-throughput experimental data, which may synergistically provide comprehensive and useful information about BC-related genes^16,17^. In our previous study^24^, we propose a method named HNP to identify BC-related genes. Although the data of three omics are integrated in HNP, the comprehensive information provided by these high-throughput data is not fully used in the algorithm^24^. In addition, more and more evidences indicate that microRNAs (miRNAs) can contribute to BC development^25^ and play the roles of suppressors or oncogenes^26^. Therefore, there is a great need to develop sophisticated methods that can efficiently integrate the heterogeneous data of both protein coding genes and non-coding miRNAs for identifying BC-related genes.

Here we propose a new method for the identification of BC-related genes by using integrative Heterogeneous Network Modeling of Multi-Omics data (iHNMMO). In iHNMMO, we make full use of known BC-related genes/miRNAs, gene expression profiles, miRNA expression profiles, CNV data, methylation data and PPIs. First, we perform a comprehensive literature curation for collecting known BC-related genes and miRNAs. Second, based on multi-omics data downloaded from TCGA, the correlational relationships of genes and miRNAs are extracted. These correlational relationships are further combined with PPIs to construct the networks of four omics. Third, the regulatory relationships between gene expression and other omics, which are used to connect the networks of different omics, are evaluated by linear regression model. Finally, based on the built heterogeneous network model, a modified propagation algorithm is implemented for the identification of BC-related genes. The comparison results show that iHNMMO achieves significantly better performance than other methods through integrating the information from different kinds of single-omics data. The predicted novel BC-related genes are also analyzed subsequently and the analysis results corroborate the superiority and effectiveness of the proposed method.

## Methods

### Multi-omics data of bladder cancer from TCGA

The multi-omics data used in this study are obtained from TCGA dataset, which provides tremendous experimental data of cancers^17^. Here the normalized data (‘level 3’ data) of four omics, i.e., CNV, gene expression, methylation and miRNA expression, are downloaded from TCGA Data Portal (https://tcga-data.nci.nih.gov/tcga). Specifically, gene expressions are derived from RNA sequencing data and the log2-transformed values are processed by quantile-normalized RSEM^27^ (RNA-Seq by Expectation Maximization). DNA methylation data used in this study are processed from Illumina HumanMethylation450 BeadChip. MiRNA expressions are log2-transformed RPMs (reads per million mapped) that calculated from sequencing data^27^. We then extract the common 377 patient samples of these four omics for follow-up studies. Since the CNV data in TCGA only contain the information of chromosome segments, we also download ‘refGene.txt’ that provides chromosomal locations of 44,914 genes from UCSC genome browser (http://genome.ucsc.edu/) and compute average CNV value of each gene accordingly. Finally, the data of four omics are normalized and transformed into four feature matrixes in which a column represents a patient sample and a row represents a gene/miRNA.

### The collection of seeds in multiple omics

We perform a comprehensive literature curation for collecting known BC-related genes and miRNAs. For genes having aberrations in methylation, CNV and gene expression, we search the keywords: “bladder cancer” AND (“methylation” OR “CNV” OR “gene expression”) on Web of Science. The selected literatures are then ranked by their citations. After manually examining the full text of the top ranked literatures, 135 BC-related genes are finally obtained, which consist of 27, 9 and 99 genes with reported aberrations in methylation, CNV and gene expression, respectively. Meanwhile, we also extract 25 known BC-related miRNAs by the keywords: “bladder cancer” AND “miRNAs”. For convenience, known BC-related genes and miRNAs are collectively called seeds.

### Pipeline of iHNMMO

**Figure 1:**
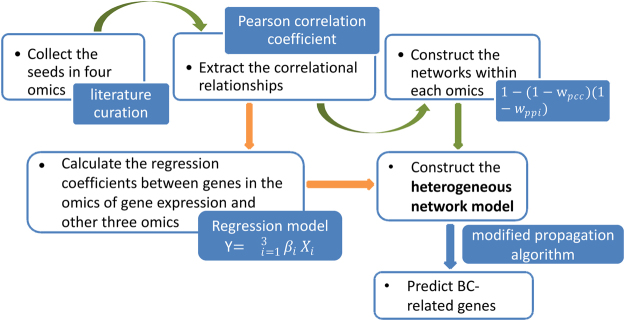
Flowchart of iHNMMO. The detailed process is described in Section “Pipeline of iHNMMO”.

The proposed method begins with seeds, which are used as true positives later. Initially, we extract correlational relationships of these seeds based on the data of four omics. Then the weighted networks of each omics are constructed through the combination of correlational relationships and PPIs. Moreover, since miRNA expression, methylation and CNV can affect expression levels of genes^18,23,26^, regulatory relationships between gene expression and the other three omics are further evaluated by linear regression model and the corresponding coefficients are utilized to weight the edges connecting the networks of different omics. In this way, the heterogeneous network model of genes is constructed, in which not only general relationships and unique relationships under BC condition, but also correlational relationships within each omics as well as regulatory relationships between different omics are considered. Finally, a modified propagation algorithm^28^ is implemented on the model to identify BC-related genes. In this process, the information flow propagates from seeds to candidate genes iteratively and a score is obtained for each candidate gene when the propagation process ends. The final score is a measurement of how much a gene can be related to BC. The overall flowchart is shown in Fig. 1.

### Heterogeneous network model for the identification of BC-related genes

As a widespread use in measuring correlational relationships^29^, Pearson correlation coefficient (PCC) is employed to reflect the correlational relationships between seeds in different omics. Specifically, for a given seed in one omics, we calculate the PCCs as well as the corresponding t-test *p-values* between this seed and other genes/miRNAs appeared in the feature matrix of this omics (see Supplementary Section 1). Afterwards, four correlation matrixes *M*_*exp*_, *M*_*cnv*_, *M*_*methy*_ and *M*_*mir*_ are built based on the correlational relationships. The element *M*(*i*, *j*) represents absolute PCC between gene/miRNA *i* and *j* in a certain omics. These matrixes are then normalized^30^ respectively to $\,\bar{{M}_{\exp }}$, $\,\overline{{M}_{CNV}}$, $\,\overline{{M}_{methy}}$ and $\,\overline{{M}_{mir}}$ as follows^30,31^:

1 $$\,\bar{{M}_{\exp }}(i,j)={M}_{\exp }(i,j)/\sqrt{{E}_{\exp }(i,i)\times {E}_{\exp }(j,j)}$$

2 $$\,\overline{{M}_{CNV}}(i,j)={M}_{CNV}(i,j)/\sqrt{{E}_{CNV}(i,i)\times {E}_{CNV}(j,j)}$$

3 $$\,\overline{{M}_{methy}}(i,j)={M}_{methy}(i,j)/\sqrt{{E}_{methy}(i,i)\times {E}_{methy}(j,j)}$$

4 $$\,\overline{{M}_{mir}}(i,j)={M}_{mir}(i,j)/\sqrt{{E}_{mir}(i,i)\times {E}_{mir}(j,j)}$$

where *E*_*exp*_(*i*, *i*), *E*_*cnv*_(*i*, *i*), *E*_*methy*_(*i*, *i*) and *E*_*mir*_(*i*, *i*) are the entities in row *i* column *i* of diagonal matrixes *E*_*exp*_, *E*_*cnv*_, *E*_*methy*_ and *E*_*mir*_, representing the sum of row *i* in *M*_*exp*_, *M*_*cnv*_, *M*_*methy*_ and *M*_*mir*_, respectively.

Besides above unique relationships under BC condition, we also take advantage of PPIs, which represent general relationships of genes. Here 4,850,628 PPIs are downloaded from STRING database^32^ (version 9.1). The redundant PPIs that do not contain the genes in the omics of gene expression are removed and 524,348 PPIs are finally extracted in this study. Likewise, these PPIs are further normalized and transformed into a PPI matrix:

5 $$\,\overline{{M}_{PPI}}(i,j)={M}_{PPI}(i,j)/\sqrt{{E}_{PPI}(i,i)\times {E}_{PPI}(j,j)}$$

Then based on the correlational relationships and PPIs above, a weighted network^33^ of the omics of gene expression is constructed as follows:

6 $${w}_{i,j}^{\exp }=1-(1-{m}_{i,j}^{Pcc})\times (1-{m}_{i,j}^{PPI})$$

where ${w}_{i,j}^{\exp }$ represents the weight of the edge in the network, ${m}_{i,j}^{Pcc}$ and ${m}_{i,j}^{PPI}$ are the elements in matrixes $\,\bar{{M}_{\exp }}$ and $\,\bar{{M}_{\exp }}$, respectively. Meanwhile, the networks of other three omics, i.e., CNV, methylation and miRNA expression, are also constructed by utilizing the correlational relationships in their omics.

**Figure 2:**
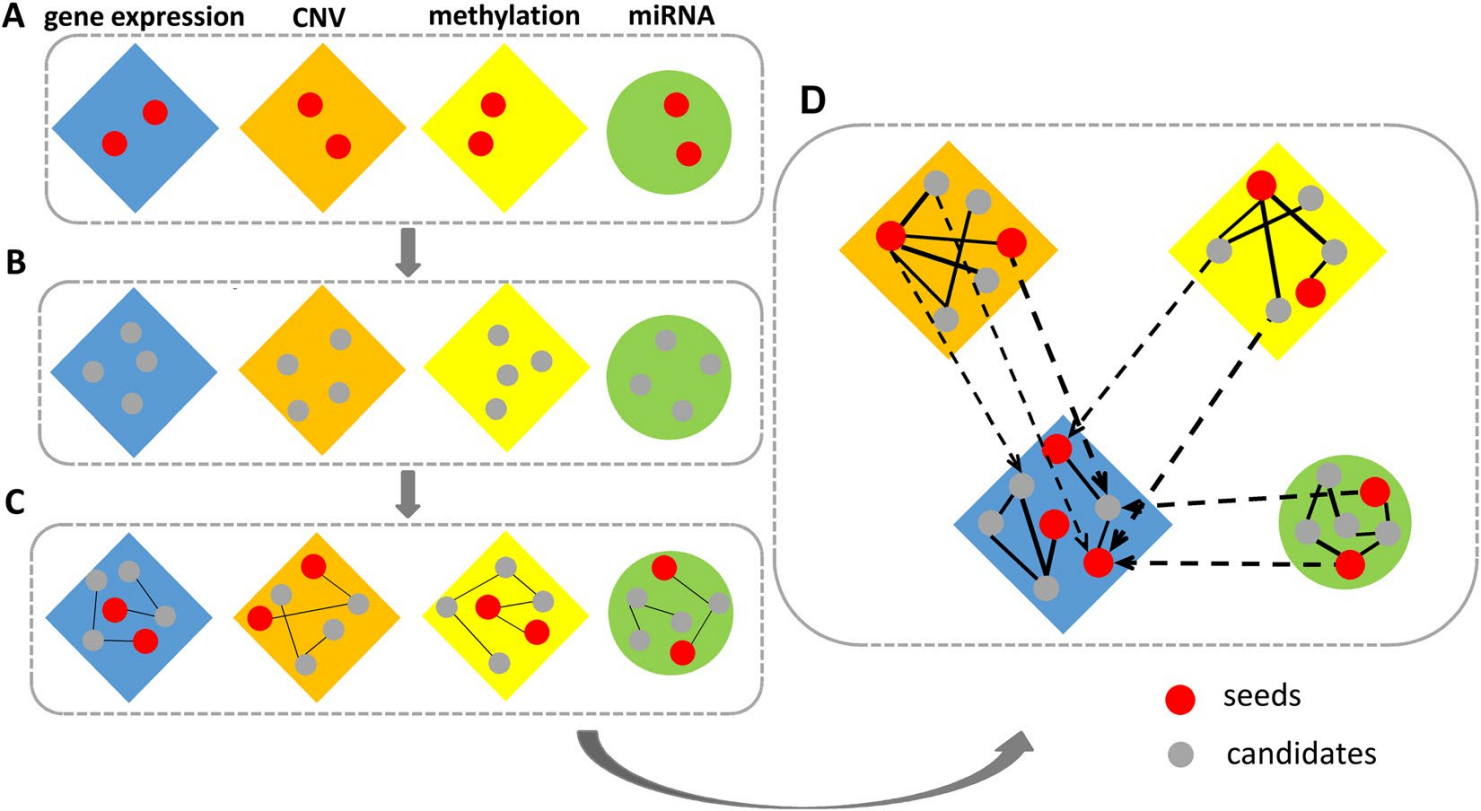
Overall process of the heterogeneous network model construction. (**A**) The collection of seeds. (**B**) Extraction of correlational relationships. (**C**) Four networks within each omics. (**D**) The heterogeneous network model.

Considering the influence on gene expression brought by miRNA expression, CNV and methylation^18,23,26^, we utilize liner regression model to evaluate the regulatory relationships between different omics. First, 17,197 regulatory relationships between genes in the omics of gene expression and miRNAs in the omics of miRNA expression are extracted from miRTarBase, which is a database of experimentally validated miRNA-gene interactions^34^. Here miRNAs that interact with a certain gene is called the miRNA regulators of this gene. Then, for a gene *i* with expression level *Y*_*i*_ (*y*_*i1*_, …, *y*_*in*_), the relationships between its CNV level *X*_*i*_^*cnv*^ (*x*_*i1*_^*cnv*^, …, *x*_*in*_^*cnv*^), its methylation level *X*_*i*_^*methy*^ (*x*_*i1*_^*methy*^, …, *x*_*in*_^*methy*^) and expression levels of its miRNA regulators *X*_*i1*_^*mir*^, …, *X*_*im*_^*mir*^ (*x*_*im1*_^*mir*^, …, *x*_*imn*_^*mir*^) (*m* and *n* are the number of miRNA regulators and the number of patient samples, respectively), are modeled using the following formula^35^ below:

7 $${Y}_{i}={\beta }_{i}^{CNV}{X}_{i}^{CNV}+{\beta }_{i}^{methy}{X}_{i}^{methy}+\sum _{j=1}^{m}{\beta }_{ij}^{mir}{X}_{ij}^{mir}+\varepsilon $$

where $\beta$ represents regression coefficient and *ε* stands for noise. Finally, we use these coefficients to connect the networks of different omics and the heterogeneous network model is constructed, in which the edges properly and comprehensively reflect the complex relationships between nodes. Besides, the normalized weight matrix $\bar{W}$ of the heterogeneous network is obtained, which denotes probability distribution of the information transition in the network. The overall process of the model construction is shown in Fig. 2.

### The modified propagation algorithm

In this study, we propose a modified propagation algorithm with resistance. Here a weighted graph model *G* = (*V*, *E*, *w*) is used to denote the heterogeneous network. In this graph model, nodes represent genes or miRNAs of four omics and edges represent the relationships between these genes or miRNAs. The weight *w* measures the confidence of the edge in the network. The goal of the algorithm is to score all candidate genes in *V* and the top-ranked genes are more probably to be BC-related genes.

First, for a node *v* ∈ *V* with direct neighbors *N*_*v*_, its prior information score *D* is calculated by following equation^33^:

8 $${D}_{v}=\{\begin{array}{ll}\quad \frac{{n}_{v}}{{N}_{v}} & {\rm{if}}\,\nu \,\mathrm{is\; non}-\mathrm{seed}\,\& \,{N}_{v}\ge \alpha \\ {e}^{{N}_{v}-\alpha }\times \frac{{n}_{v}}{{N}_{v}} & {\rm{if}}\,\nu \,\mathrm{is\; non}-\mathrm{seed}\,\& \,{N}_{v} < \alpha \\ \quad \quad {\rm{1}} & {\rm{if}}\,\nu \,{\rm{isseed}}\end{array}$$

where *N*_*v*_ is the number of neighbors for *v* and *n*_*v*_ represents the number of seeds in these neighbors. The parameter *α* is a threshold for *N*_*v*_ and it is set to 50 in this study.

To evaluate the relationship between node *v* and BC, we then introduce a probability function *S*_*v*_ based on the principle of “Guilt by Association”, which means that adjacent nodes in a network should share similar prior information and final scores^36,37^:

9 $${S}_{v}=\lambda \times (\sum _{u\in {N}_{{\rm{v}}}}{S}_{u}\times \,\overline{{W}_{{\rm{uv}}}})+(1-\lambda )\times {D}_{v}$$

where $\,\overline{{W}_{{\rm{uv}}}}$ is a component (row *u* column *v*) of |*V*| × |*V*| matrix $\bar{W}$ and *λ* ∈ (0, 1) is set to 0.2 in this study. However, when meeting a hub node, the information flow will propagate to its neighbors with the same possibility, regardless of whether these neighbors are actually related to seeds or not. In order to suppress this bias, a small amount of resistance is incorporated into the propagation process^28^, which is described as the equation below:

10 $${S}_{v}=\lambda \times (\sum _{u\in {N}_{v}}S{R}_{u{\rm{v}}})+(1-\lambda )\times {D}_{v}$$

where *SR*_*uv*_ represents the new pwwrobability for the information flow transiting from *u* to *v* with an added resistance and is formulated by:

11 $$S{R}_{uv}=\{\begin{array}{ll}0 & {\rm{if}}\,{S}_{v} < \theta {\& \max }_{t}({S}_{t}\,\overline{{W}_{{\rm{tv}}}}) < \beta \\ \max ({S}_{u}\times \,\overline{{W}_{{\rm{uv}}}}-\varepsilon ,0) & otherwise\end{array}$$

Here, *є* and *β* are respectively defined as |*V*|/|*E*|^2^ and 1/|*E*| according to^28^. Besides, *θ* is the threshold for *S*_*v*_ and we set it to 0.005. Finally, the probability function *S*_*v*_ can be further expressed in linear form:

12 $$S=\lambda \times SR+(1-\lambda )\times D$$

Since *SR* is converted from *S*, the probability function can be computed through an iterative process^15^ as follows:

13 $${S}^{t}\,:=\lambda \times f({S}^{t-1})+(1-\lambda )\times D$$

where *S* and *D* are both 1 × |*V*| matrixes, denoting the matrix of final scores and the matrix of prior information scores, respectively. Besides, ${S}^{1}:=D$. In this algorithm, the prior information is iteratively propagated from seeds to all other nodes in the heterogeneous network until the difference between *S* ^*t*^ and *S* ^*t−1*^ is sufficiently small^33^, i.e., mean square error (MSE) between *S* ^*t*^ and *S* ^*t−1*^ no larger than 1 × 10^−5^.

### The performance evaluation

To evaluate the performance of the proposed method, leave-one-out cross-validation (LOOCV) is performed in the test process. In each round, we take one seed as test data and all other seeds as training data. To prevent potential bias on seeds in network modeling, when taking a seed as test data, its correlational relationships are re-evaluated in the same way as those non-seeds. Besides, the prior information score of this seed is also recalculated by equation (8). That is, in each CV run, the topology of the heterogeneous network changes and the matrix of prior information scores *D* together with the whole weight matrix are recomputed. Especially, to impartially evaluate the performance of iHNMMO in identifying BC-related genes, we only study the scores of genes in the results. Meanwhile, the max score of a certain gene in three omics is regarded as its final score. These scores of genes are further used for performance evaluation. Seed genes and candidate genes are respectively considered as golden standard positive (GSP) and golden standard negative (GSN). Due to the fact that the top *k* ranked genes predicted by our method are defined as BC-related genes in this study, the intersections of these genes with GSN and GSP are considered as false positive (*FP*) and true positive (*TP*). After removing these intersections, the rest of GSN and GSP are referred to as true negative (*TN*) and false negative (*FN*), respectively. Then specificity (*Sp*) value and sensitivity (*Sn*) value can be obtained by the following equation:

14 $$Sp=\frac{TN}{TN+FP}\,\,Sn=\frac{TP}{TP+FN}$$

As a performance measurement, Receiver Operating Characteristic curves (ROC curves) are plotted, in which *x* axis and *y* axis represent 1-*Sp* and *Sn*, respectively. The area under this curve (AUC) is also computed. In addition, we use Rank Cutoff curves^38^ to evaluate the proportions of true positives in the top *k*% ranked genes (*k* varying from 0 to 20). Fold enrichment^30^ is also employed with the formula: fold enrichment = the number of candidate genes/2/the rank of the test gene. Here we utilize average fold enrichment of all test genes for assessment. Besides, the relationships between precision and recall with rank threshold in [100, 2000] are plotted based on the definitions:

15 $$precision=\frac{TP}{FP+TP}\,\,recall=\frac{TP}{TP+FN}$$

### Other network-based models using single-omics data

To verify the benefit from integration of multi-omics data, in this study we also examine simplified iHNMMO models with single-omics data for identifying BC-related genes, which only take advantage of the information in one omics among the multi-dimensional data of gene expression, CNV and methylation. For simplicity, these network-based models with single-omics data are thereafter named as NMSO-Expr, NMSO-CNV and NMSO-Meth, respectively.

### Data availability

The datasets and source code can be downloaded from the following URL: http://hi.ustc.edu.cn/iHNMMO/index/.

## Results

In this part, the performance of iHNMMO is evaluated systematically by comparing it with network-based models using single-omics data and other existing approaches.

### Performance comparison between iHNMMO and network-based models using single-omics data

**Figure 3:**
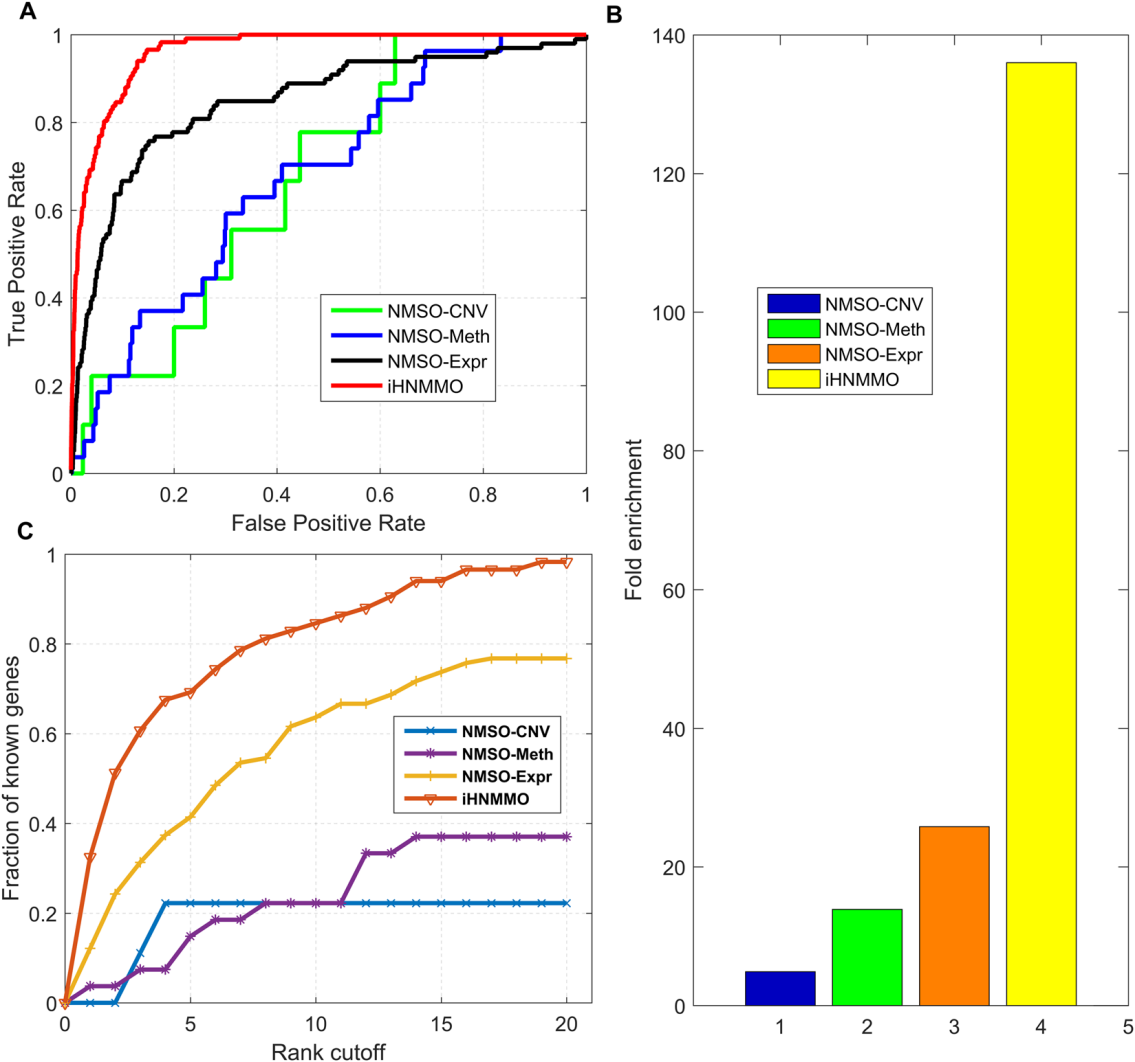
Performance comparison of iHNMMO and network-based models with single-omics data. (**A**) ROC curves. The *x* axis and *y* axis represent 1-*Sp* and *Sn*, respectively. (**B**) Fold enrichment. (**C**) Rank cutoff curves.

To verify the superiority of iHNMMO, we utilize several measurements to compare the performance of iHNMMO with network-based models using single-omics data. As shown in Fig. 3A, the ROC curve of iHNMMO is obviously above those of network-based models with single-omics data. Moreover, the AUC of iHNMMO is the largest among these methods, which is 11.3%, 28.0%, and 28.4% higher than that of NMSO-Expr, NMSO-CNV, and NMSO-Meth, respectively. Besides, at three stringent levels of *Sp*, the *Sn* values of iHNMMO are always the highest (Table 1). Specifically, at the high level of *Sp*, i.e., 99.0%, the *Sn* value of iHNMMO reaches 45.3%, which is much higher than those of other models. The huge promotion of *Sn* value corroborates the superiority of iHNMMO in improving the probability of detection. At the medium level of *Sp*, i.e., 95.0%, the *Sn* value of iHNMMO has a 29.1% growth and reaches 74.4%, while the *Sn* values of other models are 22.2%, 14.8% and 44.4%, respectively. As *Sp* level drops to 90.0%, the *Sn* value of iHNMMO rises to 86.3%, which is still higher than those of other methods.

**Table 1:** Performance comparison between iHNMMO and network-based models with single-omics data using *Sn* values at stringent levels of *Sp*.

method	**iHNMMO**	NMSO- Meth	NMSO-CNV	NMSO-Expr
*AUC*	**95**.**9%**	67.5%	67.9%	84.6%
*Sn*	**45**.**3%**	0%	3.7%	16.2%
*Sp*	99.0%			
*Sn*	**74**.**4%**	22.2%	14.8%	44.4%
*Sp*	95.0%			
*Sn*	**86**.**3%**	22.2%	22.2%	66.7%
*Sp*	90.0%			

Here *TP* and *FP* stand for true positives and false positives, *TN* and *FN* for true negatives and false negatives, respectively.

Besides, the rank cutoff curves are plotted in Fig. 3B. Similar to Fig. 3A, the curve of iHNMMO is clearly above those of network-based models using single-omics data, which indicates a better performance of iHNMMO. The fraction enlarges as the threshold increases and the curve of iHNMMO rises most rapidly when the threshold varies from 0 to 5%. For the top 5% ranked genes, the fraction of true positives predicted by iHNMMO is 69.2% (Table 2), while the fractions of the other models are all less than half. When the threshold enlarges to top 10% and 15%, the fractions of iHNMMO are 84.6% and 94.0%, respectively, both of which are still the highest among these methods. Furthermore, the fraction of iHNMMO reaches 98.3% when the threshold is 20%, which is 21.5%, 76.1% and 61.3% higher than that of NMSO-Expr, NMSO-CNV and NMSO-Meth, respectively. This phenomenon suggests that iHNMMO can always predict the largest number of seed genes with different rank cutoffs. Since the numbers of seed genes are different in different methods, we further consider the fractions of seed genes in the network and calculate the hypergeometric-test *p-values* accordingly (Table 2). The *p-values* of iHNMMO are all statistically significant (<0.05) and consistently smaller than those of network-based models with single-omics data. In Fig. 3C, the average fold enrichment of iHNMMO and network-based models with single-omics data are 136, 5, 14 and 26, respectively, indicating that iHNMMO can better identify BC-related genes with higher rank. All these results suggest that iHNMMO significantly exceeds those network-based models using single-omics data and confirm the great advantage of the heterogeneous network model that constructed by integrating multi-omics data.

**Table 2:** The fractions and corresponding *p-values* of known BC-related genes predicted by iHNMMO, NSD-CNV, NSD-Meth and NSD-Expr.

	**iHNMMO**		NMSO-CNV		NMSO-Meth		NMSO-Expr	
	fraction	*p-value*	fraction	*p-value*	fraction	*p-value*	fraction	*p-value*
Top 5%	**69**.**2%**	**1**.**3** × **10** ^**−79**^	22.2%	6.3 × 10^**−**2^	14.8%	3.3 × 10^**−**2^	41.4%	6.3 × 10^**−**28^
Top 10%	**84**.**6%**	**8**.**2** × **10** ^**−82**^	22.2%	1.7 × 10^**−**1^	22.2%	3.2 × 10^**−**2^	63.6%	5.0 × 10^**−**39^
Top 15%	**94**.**0%**	**9**.**0** × **10** ^**−83**^	22.2%	2.5 × 10^**−**1^	37.0%	3.0 × 10^**−**3^	73.7%	1.3 × 10^**−**39^
Top 20%	**98**.**3%**	**9**.**7** × **10** ^**−79**^	22.2%	3.0 × 10^**−**1^	37.0%	1.9 × 10^**−**2^	76.8%	2.9 × 10^**−**34^

### Performance comparison with existing approaches

**Figure 4:**
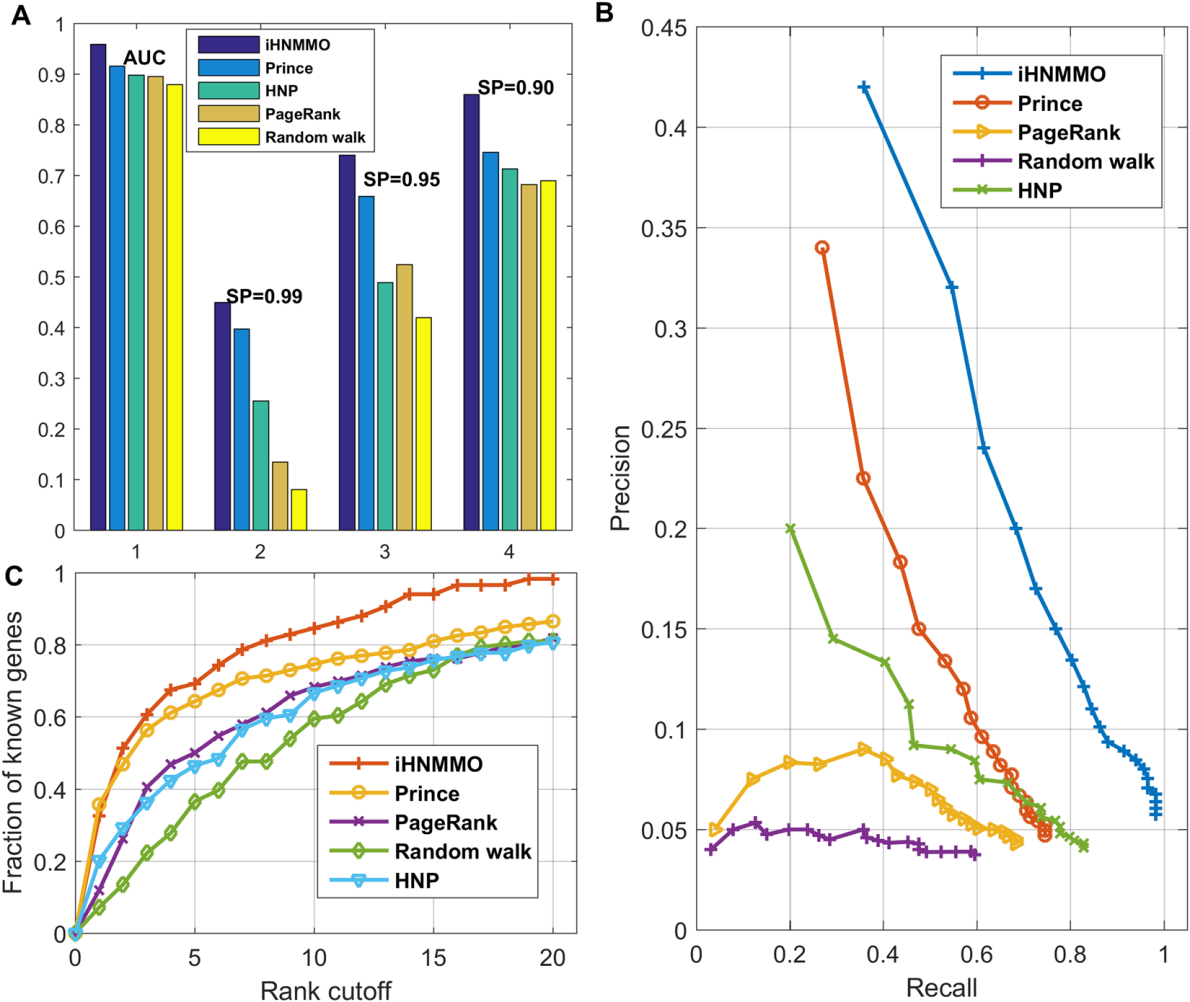
Performance comparison of iHNMMO and existing approaches. (**A)** AUC values and *Sn* values at different levels of *Sp*. (**B**) Precision-recall curves. (**C)** The rank cutoff curves. The *x* and *y* axis respectively represents the threshold and the fraction of known BC-related genes.

To perform a comprehensive comparison of the proposed method with existing approaches, we implement four network-based approaches for identification of BC-related genes: PRINCE^15^, PageRank algorithm^39^, HNP^24^ and the original random walk algorithm^14^ (see Supplementary Section 1). Their performance are also comprehensively evaluated. As shown in Fig. 4A, the AUC value of iHNMMO is 95.9%, which is 4.3%, 6.4%, 6.1% and 8.0% higher than that of PRINCE, PageRank, HNP and Random walk, respectively. In addition, at three levels of *Sp*, iHNMMO always achieves the highest *Sn* value. Specifically, when *Sp* is 99%, the *Sn* value of iHNMMO is 45% while the *Sn* values of other four approaches are 39.7%, 13.5%, 25.5% and 8%, respectively. When *Sp* level decreases to 90%, the *Sn* values of PRINCE, PageRank, HNP and Random walk rise to 74.6%, 68.2%, 71.3% and 69%, which are 11.4%, 17.8%, 14.7% and 17% lower than that of iHNMMO, respectively. These results indicate a better accuracy of iHNMMO than other four approaches. In Fig. 4B, the precision-recall curve of iHNMMO is obviously above other four curves. Within the top 100 ranked genes, the precision of iHNMMO can even reach 42%, which is 8%, 37%, 22% and 38% higher than that of PRINCE, PageRank, HNP and Random walk, respectively. At the same time, the recall of iHNMMO and other four approaches are 36%, 27.0%, 4%, 20% and 3%, respectively. The higher recall also represents the better performance of iHNMMO in retrieving known BC-related genes by ranking them into top *k*. When *k* rises to 2000, the recall of iHNMMO can even reach 98%. Besides, from the rank cutoff curves shown in Fig. 4C, we can see that iHNMMO always achieves a higher fraction of seed genes than other four approaches in the whole range. When the threshold rises to top 3%, iHNMMO can recover more than half of seed genes. All the above results of performance comparison indicate that iHNMMO remarkably outperforms PRINCE, PageRank, HNP and Random walk in identifying BC-related genes. We also respectively apply the original random walk algorithm to the heterogeneous network model and implement the modified propagation algorithm on the PPI network model. The performance of these two approaches are evaluated and compared with iHNMMO in Supplementary Section 2.

### Identifying novel BC-related genes

To analyze the predicted results of our method globally, the top 100 ranked genes that do not contain seed genes are picked up and functional enrichment analysis using DAVID are performed here. Interestingly, as shown in Table 3, functions: “GO:0042127~regulation of cell proliferation” (*p-value* = 1.5 × 10^−2^), “GO:0060548~negative regulation of cell death” (*p-value* = 1.6 × 10^−2^) and “GO:0043065~positive regulation of apoptotic process” (*p-value* = 2.6 × 10^−4^) appear in the results, which are common biological activities in human cancer^40,41^. Besides, many important pathways that related to cancer especially BC are listed in the table, e.g., “hsa04151: PI3K-Akt signaling pathway” (*p-value* = 2.8 × 10^−9^), “hsa05200: Pathways in cancer” (*p-value* = 1.0 × 10^−4^), “hsa04014: Ras signaling pathway” (*p-value* = 4.1 × 10^−4^), “hsa04350: TGF-beta signaling pathway” (*p-value* = 1.4 × 10^−3^), “hsa04010: MAPK signaling pathway” (*p-value* = 1.2 × 10^−3^), “GO:0007219~Notch signaling pathway” (*p-value* = 1.3 × 10^−2^) and “hsa04115: p53 signaling pathway” (*p-value* = 6.8 × 10^−8^). Among these pathways, some alterations of components AKT1, PTEN, TSC1 and PIK3CA in PI3K-Akt pathway of bladder cancer are observed to be remarkably related to tumor phenotype and clinical behavior^42^, Ras-mediated signaling pathway is expected to promote diagnostics and therapeutics of bladder cancer^43^, TGF-beta signaling pathway has been verified to have a possible involvement in the progression of BC^44^, new inactivating mutations of the components in Notch pathway are reported in more than 40% of BC^45^ and altered p53 pathway is expected to be an important prognostic factor on BC patient survival according to the study of^46^. All these studies indicate the potential relationships between the predicted genes and BC.

**Table 3:** Functional enrichment analysis of the top 100 ranked genes.

Category	Term	Count	*P-Value*
KEGG_PATHWAY	hsa04151:PI3K-Akt signaling pathway	19	2.8 × 10^−9^
GOTERM_BP_DIRECT	GO:0048146~positive regulation of fibroblast proliferation	5	9.2 × 10^−5^
KEGG_PATHWAY	hsa05200:Pathways in cancer	14	1.0 × 10^−4^
GOTERM_BP_DIRECT	GO:0043065~positive regulation of apoptotic process	7	2.6 × 10^−4^
KEGG_PATHWAY	hsa04014:Ras signaling pathway	10	4.1 × 10^−4^
KEGG_PATHWAY	hsa04350:TGF-beta signaling pathway	6	1.4 × 10^−3^
KEGG_PATHWAY	hsa04010:MAPK signaling pathway	9	3.8 × 10^−3^
GOTERM_MF_DIRECT	GO:0004714~transmembrane receptor protein tyrosine kinase activity	3	5.9 × 10^−3^
GOTERM_BP_DIRECT	GO:0007219~Notch signaling pathway	4	1.3 × 10^−2^
GOTERM_BP_DIRECT	GO:0042127~regulation of cell proliferation	5	1.5 × 10^−2^
GOTERM_BP_DIRECT	GO:0060548~negative regulation of cell death	3	1.6 × 10^−2^
KEGG_PATHWAY	hsa04115:p53 signaling pathway	4	3.2 × 10^−2^

Furthermore, to explore the predicted genes in detail, we list the names and the normalized scores of the top 10 ranked genes predicted by iHNMMO in Table 4. In the latest literature^47^, the first-ranked gene CCNE2 is found to be a possible prognostic marker for BC patients^47^. At the same time, another latest literature^48^ reports that FSCN1 is implicated in the pathway of hsa-miR-145-ZEB1/2-FSCN1, which is used by lncRNA-UCA1 to reinforce cell migration and invasion of bladder cancer^48^. These new discoveries are good evidence for the reliability of our results. The third ranked gene KLK3 is a member of kallikrein-related peptidases, which are expressed aberrantly in many cancers^49^ such as prostate cancer, ovarian cancer^50^ and urogenital malignancies^51^. Besides, KLK3 has been found to be related to prostate cancer in several previous studies^52,53^. From these studies, we can see that although KLK3 is not directly related to BC, the functions in other cancers may imply its potential role in BC. The top ranked miRNAs are also analyzed in Supplementary Section 2.

**Table 4:** The information of the top 10 ranked predicted genes.

Ranking	Genes	normalized scores
1	CCNE2	0.77
2	FSCN1	0.66
3	KLK3	0.63
4	FGFR4	0.63
5	CDC25A	0.62
6	CGB7	0.60
7	TFAP2A	0.59
8	WT1	0.58
9	PRDM16	0.57
10	SNAI1	0.57

## Discussion

We present a multi-step method named iHNMMO to identify BC-related genes by constructing a heterogeneous network model based on the integration of multi-omics data. Commonly, network-based algorithms for the identification of disease-related genes are motivated by the discovery that genes closing to one another are more likely to lead to the same or similar diseases^15^. Therefore, whether the network model can reflect the relationships of genes suitably is critical to the method. In this study, we address this issue by integrating multi-omics data. According to the information provided by the data of methylation, miRNA expression, gene expression and CNV, we obtain both regulatory and correlational relationships of genes, which are further used to build and combine the networks of four omics. Besides, to fully reflect general relationships and unique relationships under BC condition, not only the correlations calculated by statistical analysis, but also PPIs downloaded from well-established database are utilized to generate the correlational relationships between genes. Thus, the heterogeneous network model that contains comprehensive information for the identification of BC-related genes is set up, which may be the most important factor leading to the success of iHNMMO. In addition, another factor that contributes to the superiority of iHNMMO is the modified propagation algorithm implemented on the model, which can score and rank candidate genes precisely. It is also important to note that the heterogeneous network model and propagation algorithm should be integrated properly to make sure their advantages could be fully used. For example, although our previous method HNP^24^ utilizes the data of three omics, it does not achieve a comparable performance with iHNMMO. This may be due to the fact that the comprehensive information provided by multi-omics data is only used at the beginning of HNP, i.e., the initialization of propagation, which does not sufficiently promote the whole process of propagation.

Although our method achieves an excellent performance in identifying BC-related genes, it will be better to utilize independent datasets to facilitate a fair performance assessment. However, we cannot perform more rigorous evaluation because of the lack of parallel data of complete four omics. Actually, this issue also occurs in many computational studies of multi-omics data^36,54–56^ in cancer. The insufficiency of data also leads to some limitations in the generalization of iHNMMO to other diseases. For example, since the heterogeneous network is constructed based on seeds, iHNMMO cannot be generalized to those diseases that have no known disease-related genes. In this case, other information such as the similarities between diseases will be introduced to the method to make comparison of candidate genes and the genes that are known to be related to similar diseases. Similarly, iHNMMO cannot be performed on the researches where multi-omics data are incomplete even unavailable. However, high-throughput technologies with reduced cost such as next generation sequencing and microarrays develop rapidly, and many researches of multi-omics data are underway now. It is believed that we can obtain more comprehensive data of different omics in the future. Moreover, the functions of long non-coding RNAs (lncRNAs) are explored in more and more cancer studies^29,33^ and these information should be incorporated properly into the heterogeneous model to reflect the molecular mechanism of disease better. Despite the difficulties listed above in the generalization of iHNMMO, our method can still be well applied to identify other disease-related genes as long as relevant data meets the requirement in this study. Here we take glioblastoma (GBM) as an additional example and the known GBM-related genes and multi-omics data of GBM processed in our previous study^57^ are utilized. The results of performance evaluation are shown in Supplementary Section 2, which indicate the good generalization ability of iHNMMO.

## Electronic supplementary material

Supplementary Data
