# Supplementary material for: Discovery of Bladder Cancer-related Genes Using Integrative Heterogeneous Network Modeling of Multi-omics Data
Source: Sci Rep. 2017 Nov 15;7:15639. doi: 10.1038/s41598-017-15890-9 (PMC5688092; doi:10.1038/s41598-017-15890-9)
Supplement: Supplementary file 1 — Supplementary Data [file 41598_2017_15890_MOESM1_ESM.pdf]

Discovery of Bladder Cancer-related Genes Using Integrative Heterogeneous Network Modeling of Multi-omics Data

——Supplementary Information

Chen Peng[1, 2] Ao li[1, 3*] and Minghui Wang[1, 3]

[1] School of Information Science and Technology, University of Science and Technology of China, Hefei, AH230027, China

[2] Institute of Machine Learning and Systems Biology, College of Electronics and Information Engineering, Tongji University, Shanghai, 201804, P.R. China

[3] Centers for Biomedical Engineering, University of Science and Technology of China, Hefei, AH230037, China

aoli@ustc.edu.cn

1. Supplementary Methods

1.1. Heterogeneous network model for BC-related gene identification

As a widely used measurement of correlational relationships[1], Pearson correlation coefficient (Pcc) is employed to reflect the correlational relationships between the seeds in the omics of gene expression, CNV, methylation and miRNA expression, respectively. For a seed in a certain omics, we calculate the Pcc values and corresponding t-test *p-values* between this seed and all other genes listed in this omics. Only gene pair with *p-value* no larger than 0.01 and absolute Pcc value ranked in the top 0.5 percent[1] is kept as the correlational relationship of this seed. Then we extract the correlational relationships of the seeds in the omics of miRNA expression similarly, except that the threshold of Pcc is set to top 5 percent since the number of miRNAs is small. The neighbors of these seeds in the correlational relationships are regarded as candidates and the correlational relationships between them are also explored in the same way as the seeds. Totally we get 9089 genes and 426 miRNAs in the omics of gene expression and miRNA expression, respectively. For all 9,089 genes in the omics of gene expression, there are 741 and 149 genes lacking the information of CNV and methylation, respectively. Therefore, these genes are abandoned and we regard the remaining 8,348 and 8,940 genes as the final elements in the omics of CNV and methylation, which include 9 and 27 seeds, respectively. The correlational relationships of these genes in each omics are extracted with the same method as mentioned before. Then four correlation matrixes M_{exp}, M_{cnv}, M_{methy} and M_{mir} are built based on the

correlational relationships:

$$\overline{M_{\exp}}(i,j)=M_{\exp}(i,j)\Big/\sqrt{E_{\exp}(i,i)\times E_{\exp}(j,j)} \quad (1)$$

$$\overline{M_{CNV}}(i,j)=M_{CNV}(i,j)\Big/\sqrt{E_{CNV}(i,i)\times E_{CNV}(j,j)} \quad (2)$$

$$\overline{M_{methy}}(i,j)=M_{methy}(i,j)\Big/\sqrt{E_{methy}(i,i)\times E_{methy}(j,j)} \quad (3)$$

$$\overline{M_{mir}}(i,j)=M_{mir}(i,j)\Big/\sqrt{E_{mir}(i,i)\times E_{mir}(j,j)} \quad (4)$$

1.2. Details of the performance comparison with existing approaches

To perform a comprehensive comparison of the proposed method with existing approaches, we implement four network-based approaches for disease-related gene identification: the original random walk algorithm[2], PRINCE[3], PageRank algorithm[4] and HNP[5]. Here we download totally 4,850,628 protein-protein interactions (PPIs) from STRING database[6] and transform these PPIs into matrix W_{PPI}. The matrix is also normalized as follows:

$$\overline{M_{PPI}}(i,j)=M_{PPI}(i,j)\Big/\sqrt{E_{PPI}(i,i)\times E_{PPI}(j,j)} \quad (5)$$

Then the weighted PPI network is constructed. We further implement the first three approaches on this PPI network and their performance are evaluated by treating all BC-related genes in the omics of gene expression, CNV and methylation as true positives. HNP is implemented and evaluated in the same way as we described in[5].

2. Supplementary Results

2.1. Supplement of the performance comparison with existing approaches

To show the advantage of the modified propagation algorithm, we also implement the original random walk algorithm on the heterogeneous network model in this study and compare its performance with iHNMMO by ROC curves, precision-recall curves and rank cutoff curves with the threshold varying from 200 to 10000. The results are depicted in Figure S1 and the better performance of iHNMMO indicates the superiority of the heterogeneous network model, which is based on the integration of multi-omics data. Besides, we also implement the modified propagation algorithm on the PPI network and the performance comparison with iHNMMO is shown in Figure S2. We can see that the performance is obviously developed by using the modified propagation algorithm.

2.2. Generalization of iHNMMO to GBM

We take GBM as an example to show the generalization ability of iHNMMO to other diseases. Here we utilize the known GBM-related genes and multi-omics data of GBM that processed in our previous study[7]. As shown in Figure S3, the AUC value of our method reaches 94.2% and the *Sn* values at three levels of *Sp* are all high. Especially, when *Sp* is 99.0%, our method can still achieve a high *Sn* value. These results of performance evaluation indicate the good generalization ability of our method to other diseases.

2.3. Analysis of top ranked miRNAs

We extract target genes of the top 10 ranked miRNAs from the heterogeneous model (Supplementary Table S1). Specifically, one third of the target genes of the first ranked miRNA mir-138-2 are seed genes, which means that more information can be directly propagated from seed genes to mir-138-2. To further explore how iHNMMO helps detect mir-138-2, the intermediate results of the algorithm are shown in Figure S4. In detail, Figure S4A shows the prior information of mir-138-2 and another 10 randomly chosen miRNAs that are not ranked in top 10, *i.e.*, the numbers of seeds connecting to them in the heterogeneous network. It is obvious that mir-138-2 has more seed neighbors, which means more information can be directly propagated from seeds to mir-138-2. The weights of the associations between mir-138-2 and its neighbors are exhibited in Figure S4B, in which some associations are relatively larger such as HIF1, TERT and ZEB2. In addition, Figure S4C indicates that mir-138-2 has a significantly larger number of neighbors in the heterogeneous network. Based on the prior information in Figure S4A and S4C, the learned local models of these miRNAs are shown in Figure S4D, which represent their prior information scores. Using these models, iHNMMO successfully retrieves mir-138-2 based on the transition probabilities, *i.e.*, the edge weights in the heterogeneous network. In the same manner, other novel BC-related genes/miRNAs can be discovered. The interactions between mir-138-2 and its target genes are shown in Figure S5.

Reference

1 Liao, Q. *et al*. Large-scale prediction of long non-coding RNA functions in a coding–non-coding gene co-expression network. *Nucleic acids research* **39**, 3864-3878 (2011).

2 Köhler, S., Bauer, S., Horn, D. & Robinson, P. N. Walking the interactome for prioritization of candidate disease genes. *The American Journal of Human Genetics* **82**, 949-958 (2008).

3 Vanunu, O., Magger, O., Ruppin, E., Shlomi, T. & Sharan, R. Associating genes and protein complexes with disease via network propagation. *PLoS Comput Biol* **6**, e1000641 (2010).

4 Brin, S. & Page, L. Reprint of: The anatomy of a large-scale hypertextual web search engine. *Computer Networks* **56**, 3825-3833 (2012).

5 Peng, C., Li, A., Feng, H. & Wang, M. in Natural Computation, *Fuzzy Systems and Knowledge Discovery (ICNC-FSKD), 2016 12th International Conference on*. 1396-1401 (IEEE).

6 Franceschini, A. *et al*. STRING v9. 1: protein-protein interaction networks, with increased coverage and integration. *Nucleic acids research* **41**, D808-D815 (2013).

7 Peng, C., Shen, Y., Ge, M., Wang, M. & Li, A. Discovering key regulatory mechanisms from single-factor and multi-factor regulations in glioblastoma utilizing multi-dimensional data. *Molecular BioSystems* 11, 2345-2353 (2015).

Supplementary Figures

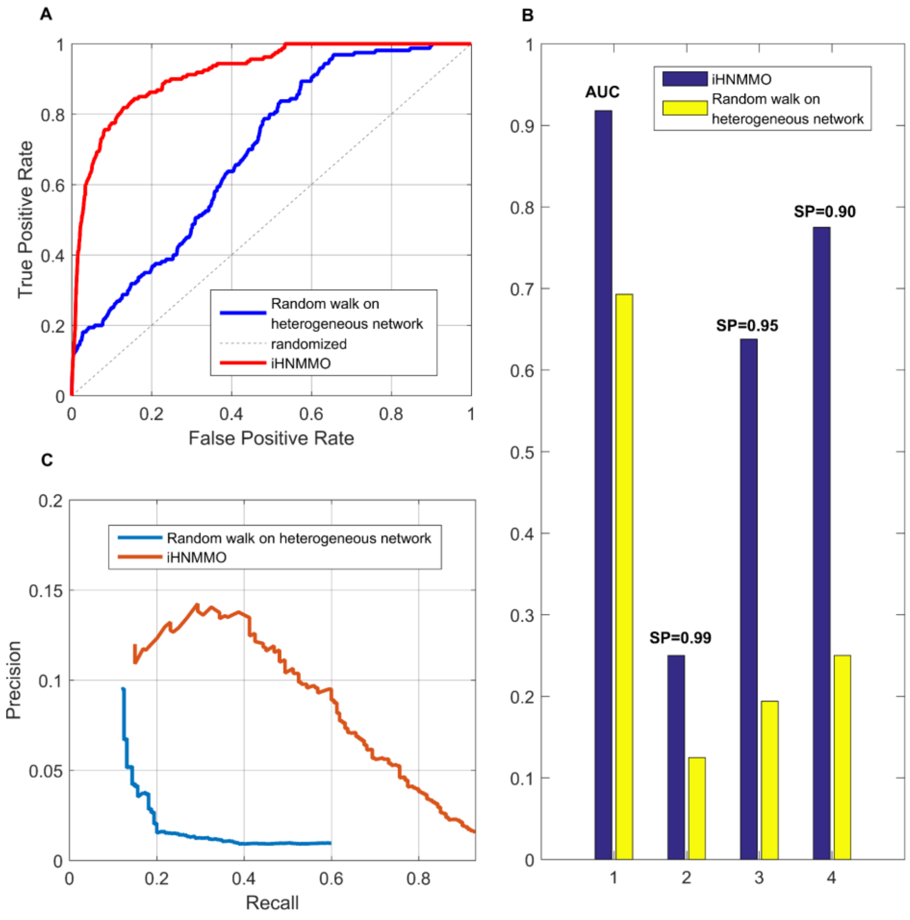

Fig. S1. Performance comparison of iHNMMO and random walk algorithm on heterogeneous network model. **A**. Roc curves. The *x* axis represents 1-*Sp* and *y* axis represents *Sn*. **B**. The rank cutoff curves. The x axis represents the threshold and y axis represents the fraction of known BC-related genes. **C**. Precision-recall curves.

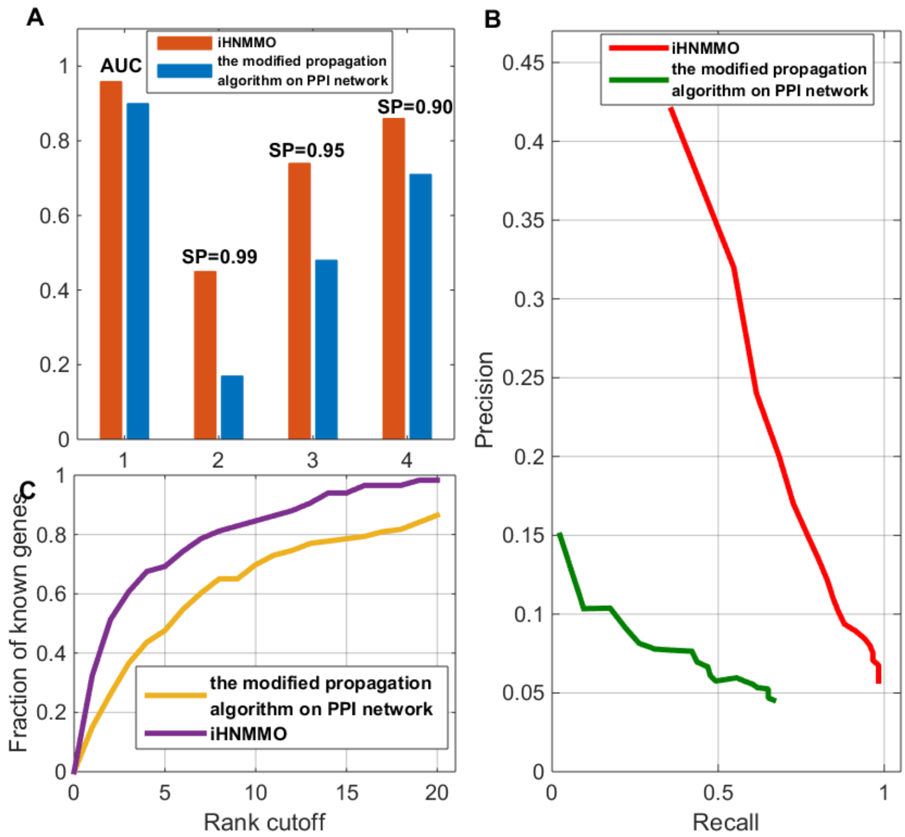

Fig. S2. Performance comparison of iHNMMO and the modified propagation algorithm on PPI network. **A**. AUC values and *Sn* values at stringent levels of *Sp*. **B**. Precision-recall curves. **C.** The rank cutoff curves. The *x* axis represents the threshold and *y* axis represents the fraction of known BC-related genes.

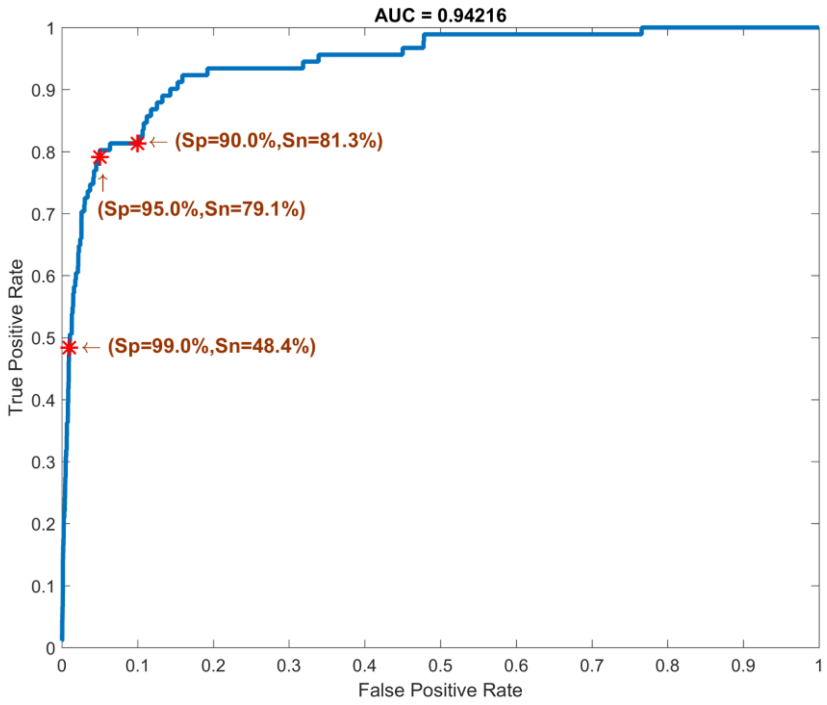

Fig. S3. The ROC curve of iHNMMO with GBM data. The points marked with red star are *Sn* values at stringent levels of *Sp*.

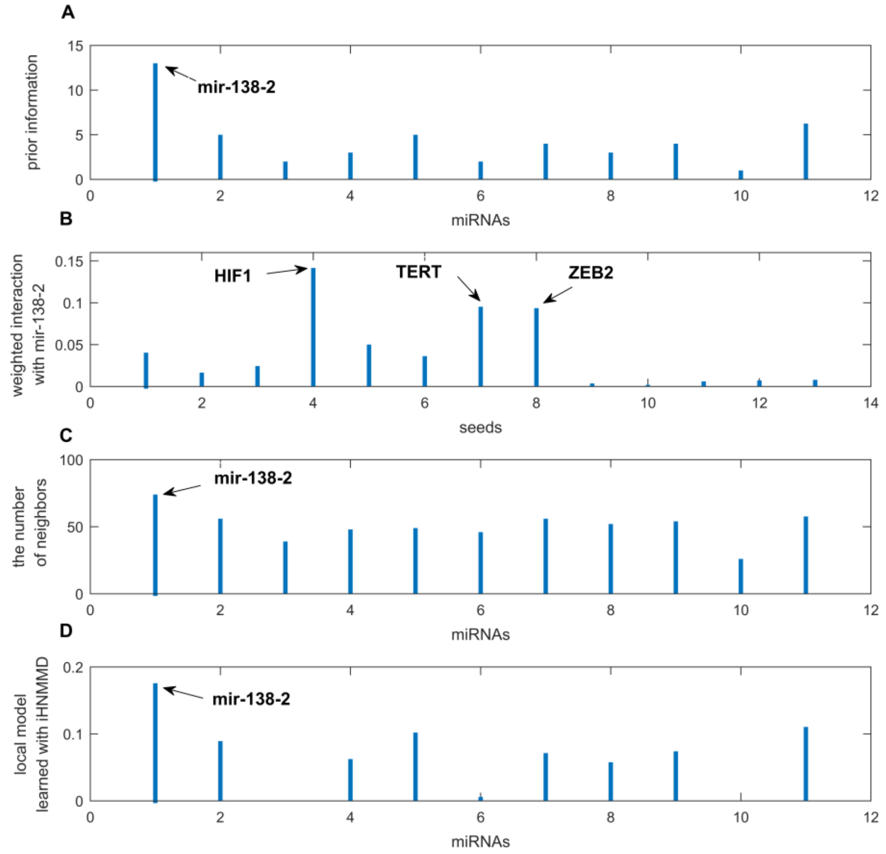

Fig. S4. The process of how iHNMMO helps detect novel BC-related genes/miRNAs. A. The prior information of mir-138-2 and another 10 randomly chosen miRNAs that are not ranked in top 10, i.e., the numbers of seeds connecting to them in the heterogeneous network. **B.** The weights of the edges between mir-138-2 and seeds. **C.** Neighbor numbers of the 11 miRNAs. D. Local models of the 11 miRNAs.

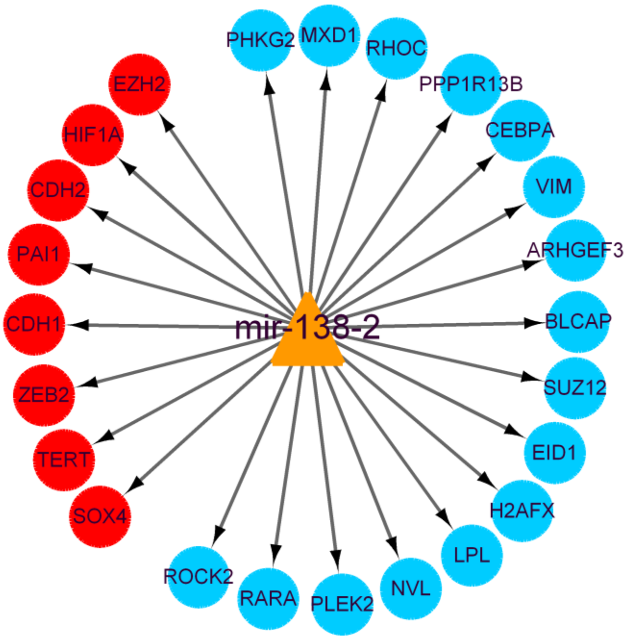

Fig. S5. The interactions between mir-138-2 and its target genes. The round nodes represent target genes and red nodes represent seed genes.

Supplementary Tables

miRNAs	Target genes
mir-138-2	ARHGEF3, BLCAP, **CDH1**, **CDH2**, CEBPA, EID1, **EZH2**, H2AFX, **HIF1A**, LPL, MXD1, NVL, PHKG2, PLEK2, PPP1R13B, RARA, RHOC, ROCK2, **PAI1**, **SOX4**, SUZ12, **TERT**, VIM, **ZEB2**
mir-205	CTGF, CYR61, DDX5, E2F1, **ERBB2**, ERBB3, IL24, LRP1, LRRK2, SIGMAR1, **TP73**, ZEB1, **ZEB2**
mir-138-1	ARHGEF3, BLCAP, **CDH1**, **CDH2**, CEBPA, EID1, **EZH2**, H2AFX, **HIF1A**, LPL, MXD1, NVL, PHKG2, PLEK2, PPP1R13B, RARA, RHOC, ROCK2, **SERPINE1**, **SOX4**, SUZ12, **TERT**, VIM, **ZEB2**
mir-203	None
mir-135b	IBSP, **KLF4**, MAFB
mir-10b	ACLY, AP3M1, ARFGEF1, BCL2L11, C6orf106, CDK12, CDK2, **CDKN1A**, **CDKN2A**, COL6A2, CSNK2A1, CTBP1, DDHD2, DMWD, ERLIN1, EXTL3, FEM1B, GBAS, GK, HIST1H2AL, HK1, HOXD10, INHBA, KIAA0100, **KLF4**, LEMD3, LMF2, LRP3, LRRC59, MAPRE1, NCOR2, NR4A3, NRP2, PABPC1, **PAX6**, PIK3CD, PLA2G4F, POLR2A, PPP1R13B, PRPF8, PSMD13, PTPRT, RALBP1, **RBM17**, RPS8, RPS9, **SDC1**, SLC25A5, SLC2A3, **TP53**, TRA2B, TSPAN33, UBE2Z, **UHRF1**, UROD, USP3, ZNF318
mir-199b	**ERBB2**, HES1, **HIF1A**
mir-490	ERGIC3
mir-642a	MDN1
mir-143	AKT1, **BCL2**, COL1A1, DNMT3A, FHIT, FNDC3B, FSCN1, KRAS, **MDM2**, MMP13, **SERPINE1**

Table S1. The target genes of top 10 ranked miRNAs. The bold gene names represent seeds.
